# Digital Image Filtering Optimization Supporting Iberian Ham Quality Prediction

**DOI:** 10.3390/foods9010025

**Published:** 2019-12-25

**Authors:** Francisco Perán-Sánchez, Salud Serrano, Eduardo Gutiérrez de Ravé, Elena Sánchez-López, Ana Cumplido, Francisco J. Jiménez-Hornero

**Affiliations:** 1 Department of Food Hygiene and Technology, University of Córdoba, Campus Rabanales, Edif. Darwin, Anexo, 14071 Cordoba, Spain; franperan@hotmail.com (F.P.-S.); ana.cumplido.calleja@gmail.com (A.C.); 2 Department of Graphic Engineering and Geomatics, University of Córdoba, Campus Rabanales, Edif. Mendel, 14071 Córdoba, Spaing02saloe@uco.es (E.S.-L.); ir2jihof@uco.es (F.J.J.-H.)

**Keywords:** Iberian ham, high-pass filter, image analysis, fatty infiltration, manual slicing, multifractal analysis

## Abstract

Digital images of food for later analysis tend to be heterogeneous in terms of color and luminosity. Improving these images by using filters is necessary and crucial before further processing. This paper compares the non-use of filters and the use of high-pass filters in the images of hand-cut Iberian ham that will be used in a multifractal analysis for the study of fat and its infiltration. The yielded results show that with the use of a high-pass filter, more accurate fractal dimensions were obtained, which can be featured in predictive techniques of Iberian ham quality.

## 1. Introduction

The Iberian pig is a Spanish breed of great economic importance that is raised under different rearing systems. The dry-cured ham from this Iberian pig is classified in different categories, such as acorn or feed, depending on the feeding regimen that is provided. The main contributors to the quality characteristic and intense flavour of this product are the (i) meat quality, (ii) rearing system, mainly during the final fattening period, called “montanera”, during which the pigs are fed with pasture and acorns from the Quercus genus, the predominant species in the “Dehesa” (Mediterranean silvopastoral system), (iii) age of animals, (iv) pig genotype, and (v) conditions of the ripening process [1].

Acorn-fed ham comes from Iberian pigs raised on free-range farms with a diet of acorns and grass, while feed-fed ham comes from pigs raised on confined farms and fed with concentrated feed. It is regulated by the Spanish government (Royal Decree 4/2014) [2].

Particularly, pure Iberian pigs reared outdoor and fed on “montanera” provide the highest sensory quality and are the most highly appreciated by consumers [3]; therefore, their prices are consistently higher than those of other dry-cured meat products. Furthermore, Diaz-Caro et al. [4] stated that this type of feed is the most preferred attribute by consumers, in line with the sensory analysis.

Intramuscular fat (IMF), recognized by the consumer as marbling, is the most important characteristic for the Iberian ham quality. Marbling is a visual attribute of meat that affects the acceptability and palatability and that is defined as the amount and spatial distribution of the visible fat within the muscle [5]. Marbling can be assessed visually, or measured using image analysis, while chemical analyses are unable to supply the information on the spatial distribution and characteristics of streaks of fat [6].

Image analysis has numerous applications to the food industry, such as classifying pork hams, evaluating the quality of cold meat, assessing the tenderness of beef carcasses, controlling the freshness of gilthead sea bream based on gill and eye color changes, and automatic fishbone detection [7,8,9,10,11,12]. Visible image analysis is one of the most accessible techniques, from which many studies have offered successful results in diverse research areas on versatility and reduced cost possibilities.

The general procedure in most of these studies comprises three main steps: image acquisition, image analysis, and data analysis. Image acquisition requires the scrupulous design of the image capturing system and careful operation to obtain high-quality digital images. Image analysis includes numerous algorithms and methods available for classification and measurement. The automatic colour measurement using computer vision has the advantages of a superior speed, consistency, accuracy, and cost-effectiveness, and therefore, not only can it optimize the quality inspection, it also helps in reducing human inconsistency and subjectiveness [13].

Filtering is often considered as a prerequisite to many image analyses tasks. Digital image filtering refers to the modification of the pixels in an image based on some function of its neighboring pixels [14]. By adjusting the radius of the analyzed pixels according to the pixel on which the filtering is done, different results will be obtained.

Filtering can be carried out on the frequency and/or space domains. If it is about the frequency domains, they amplify or attenuate certain frequency components in an image. If the filter attenuates certain high frequencies of an image, it is commonly known as a low-pass filter. These filters provide smoothness to an image by suppressing high-frequency components. In contrast, high-pass filters suppress the low-frequency components of an image by enhancing its edges [14].

It has been shown that high-pass filters are good homomorphic filters for image enhancement [15]. The Gaussian filter is widely used in various fields such as medical X-ray images [16,17,18] and aquatic images [19].

Image analysis has recently been associated with fractal analysis, that is, a group of calculated algorithms used to study and characterize highly complex systems with chaotic structures to detect measurable patterns that can offer valuable information from a given sample [20]. The extraction of textural information from images is very common for exploring parameters related to food quality. The fractal concept studies the degree of self-similarity found in a structure at all scales. Mainly, the use of fractals allows the identification of recurring patterns. In recent years, there has been growing interest in the use of fractal analysis techniques [21]. Some examples are the characterization of fatty infiltration in Iberian and white pork sirloins, color changes on the surface of fresh cut meat, evaluations of the effects of frozen storage on the tilapia microstructure [22,23,24], quality of meat [25], crystallizing food systems [26], attributes of pork loin [21], and characterization of the pork and salmon composition [20].

The combination of digital food image analysis (DFIA) and predictive techniques to assess meat quality is arousing interest [27] because it offers some advantages over the sensory analysis, such as being non-destructive, less time-consuming, and low cost [28]. The first steps that have been taken to predict pork meat by applying data mining (Iberian ham [29,30] and loin [21,31,32]) and machine learning (marbling [33,34]) are recent. Pork meat quality prediction improves when the fractal analysis framework is taken into account [21,32]. In this sense, it has been shown that Iberian pork sirloin and ham have a fat connective tissue whose distribution can be described by means of some fractal dimensions [24,35]. As a consequence, the multifractal analysis can be regarded as a source of information for the databases used by pork meat quality prediction techniques.

In order to obtain suitable information based on the multifractal parameters, it is necessary to have precise and quick DFIA methods. Thus, it is necessary to increase the role of automated operations to obtain more accurate results. Previous studies, such as Serrano et al. [24,35], proposed a DFIA based on human intervention by manually setting some image acquisition parameters. This fact supposed a potential source of errors that forced multiple revisions to avoid them, with the consequent cost in time. With the aim of overcoming this drawback, some alternatives were explored here in order to introduce a methodology that involves less human intervention.

## 2. Materials and Methods

### 2.1. Ham Samples

For the study, a total of 121 samples of ham (hand-cut slices) were used, classified in Table 1.

The samples were donated by the company COVAP (Córdoba, Spain) in vacuum containers. For each designation, two different batches of samples were used.

Due to the complexity of taking pictures of complete slices, a different number of samples were made from each package, depending on the possibility of taking full slices.

It should be mentioned, as this is important for the study, that the samples were not taken from the same areas of the ham, so that in the same package slices could be taken from different muscles of the leg of the pig, with the consequent heterogeneity in the color of the muscle fibers and the amount of infiltrated fat and accumulated fat clusters.

### 2.2. Image Acquisition

For the acquisition of the images, the method proposed by Serrano et al. [24,35] and based on Valous et al. [36] was used.

The system consists of a cubicle with four Osram fluorescent light sources of 36 w and a color temperature of 5400 K, with an inclination of 60° on a surface with a dark matte cardboard where the samples are placed. The light source must be turned on 30 min before taking pictures to reach the correct color temperature.

The system was completed with a Nikon D60 camera with an 18–55 mm lens placed vertically about 15 cm above the black cardboard. The images were taken using standardized parameters to avoid differences in light and color in the samples. The parameters that gave the best results when taking the images, in terms of the sharpness of the images and homogeneity of light and color, were the following: image size 3872 × 2592 pixels in JPEG format, 36 mm zoom, focal length of 26 mm, speed of 1/60 s shot, f/8 aperture, ISO 200, without flash, and white balance adjusted by gray letter with 18% reflectance.

### 2.3. Image Processing

Photoshop CC 2018 software and Matlab R2019a software were used to process the images.

First, the method used by Serrano et al. [24,35] was carried out by using Photoshop to cut the 512 × 512 pixel images of the manually established areas.

**Figure 1:**
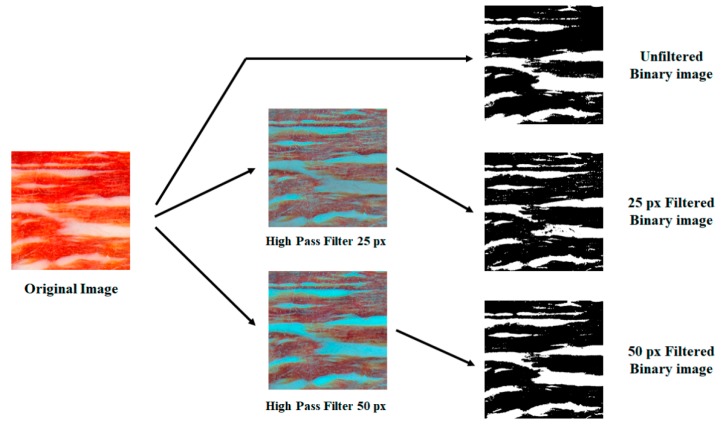
The steps to obtain binary images from the original 512 × 512 pixels image.

**Figure 2:**
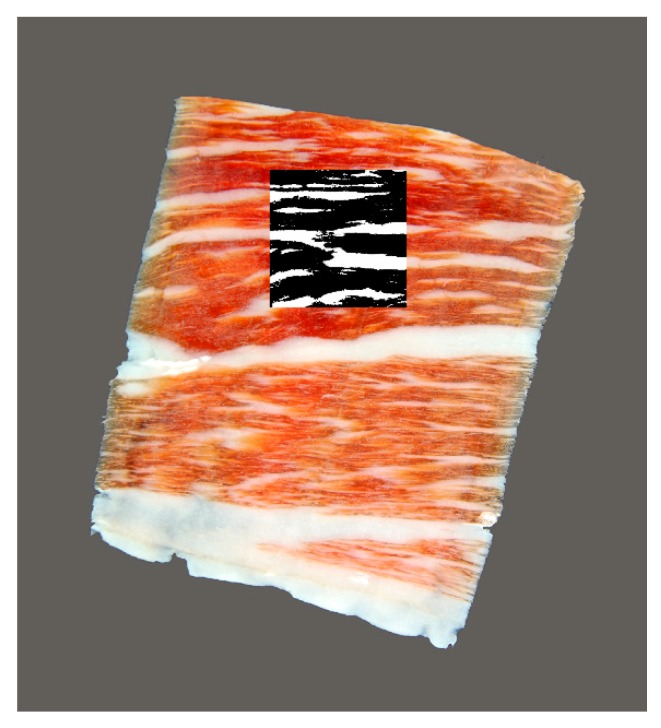
Representation of the original image, and square area of 512 pixels selected and processed to black (lean meat) and white (fatty infiltration) for analysis.

Subsequently, the squared color images were transformed into black and white using three methods to be compared (Figure 1). Then, they were transformed into black and white binary images via a manual threshold set at 175 through which the red colors, belonging to lean tissue, were transformed into black, and through which the white and yellow ones, belonging to fatty tissue, were transformed into white. Afterwards, the images were processed through a high-pass filter to overcome the problems of heterogeneity in the color of the slices of ham. Two radii of the high-pass filter were established for comparison, 25 and 50 pixels, and were superimposed on the original image. Subsequently, the images were transformed into black and white images setting a threshold of 150. Figure 2 shows an original image of the sample and the quadrate white and black region.

Once the black and white images were obtained by both methods, the images were finally transformed using Matlab into a binary code of three columns, which indicates the horizontal position, vertical position, and pixel color (“1” white color for fat; “2” black color for muscle). This data file was the one used for the performance of the multifractal analysis.

## 3. Results and Discussion

The multifractal nature of the Iberian ham samples was exhibited for the three image processing methods considered here. Thus, the next step was to check the accuracy of these methods by comparing the values obtained for the following fractal dimensions: *D*_0_, box-counting dimension or fractal dimension, related to geometric patterns but insensitive to density distribution. *D*_1_, information or entropy dimension, linked to the uniformity in the measure distribution. *D*_2_, correlation dimension, indicating the pattern complexity. More details on the multifractal sandbox method that was performed to yield these fractal dimensions can be found in Serrano et al. [24,35] and references cited in these works.

Table 2 lists the fractal dimensions’ mean values and their standard errors for each method used. The data were analyzed by using SPSS Statistics (v.26) software (IBM Corp., Armonk, NY, USA). 

There are no rules to set the acceptable standard error values when determining fractal dimensions [36]. Although those found in this work are relatively high, they are suitable because all of them are lower than 0.1 [37]. Each fractal dimension exhibits a clear ascending or descending trend following the order A100IH (Acorn 100% Iberian Ham), AIH (Acorn Iberian Ham), FPIH (Feed/Pasture Iberian Ham) and FIH (Feed Iberian Ham) for the three methods.

**Figure 3:**
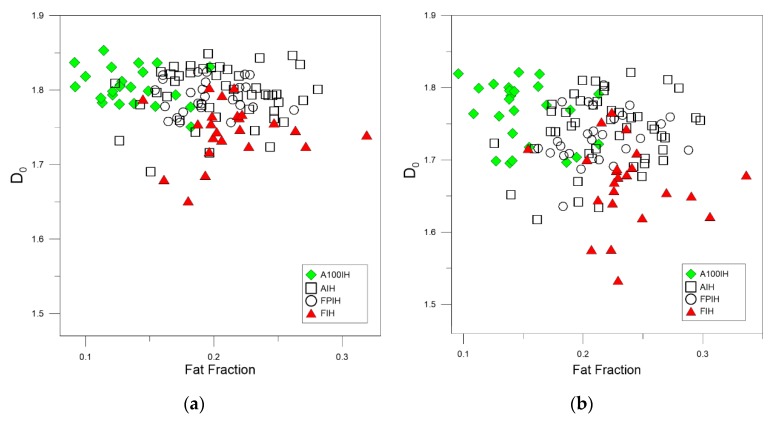

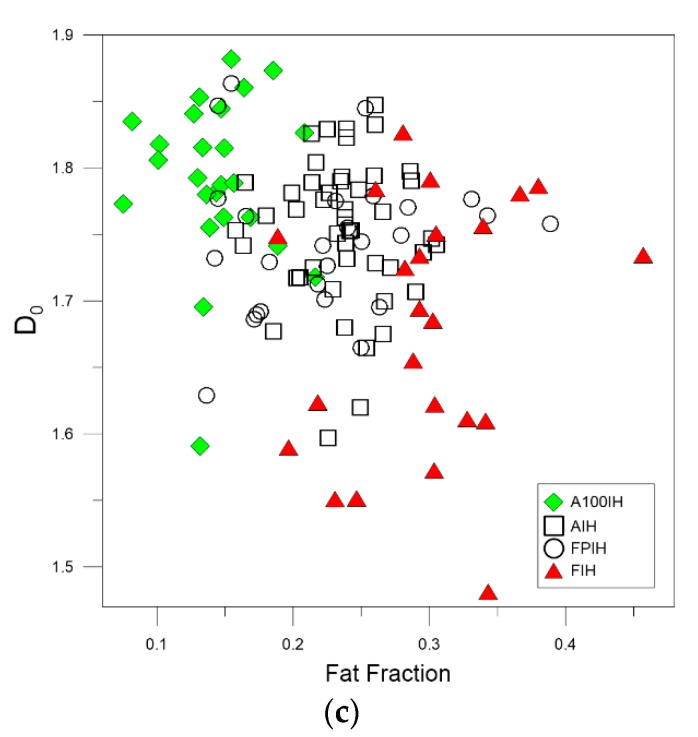
Scatter plots depicting the relationships between the box-counting dimension (or fractal dimension), *D*_0_, and the fat fraction found for each ham designation when (**a**) high-pass filter 25 pixels (**b**) high-pass filter 50 pixels (**c**) unfiltered images are used. A100IH: Acorn 100% Iberian Ham, AIH: Acorn Iberian Ham, FPIH: Feed/Pasture Iberian Ham, FIH: Feed Iberian Ham.

**Figure 4:**
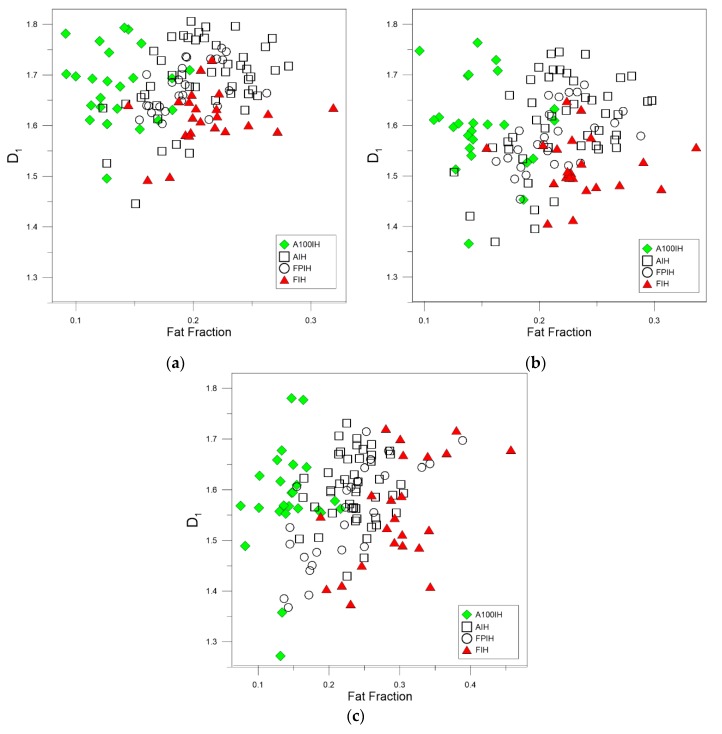
Scatter plots showing the associations between the information or entropy dimension, *D*_1_, and the fat fraction obtained for each ham designation when (**a)** high-pass filter 25 pixels (**b**) high-pass filter 50 pixels (**c**) unfiltered images are used.

**Figure 5:**
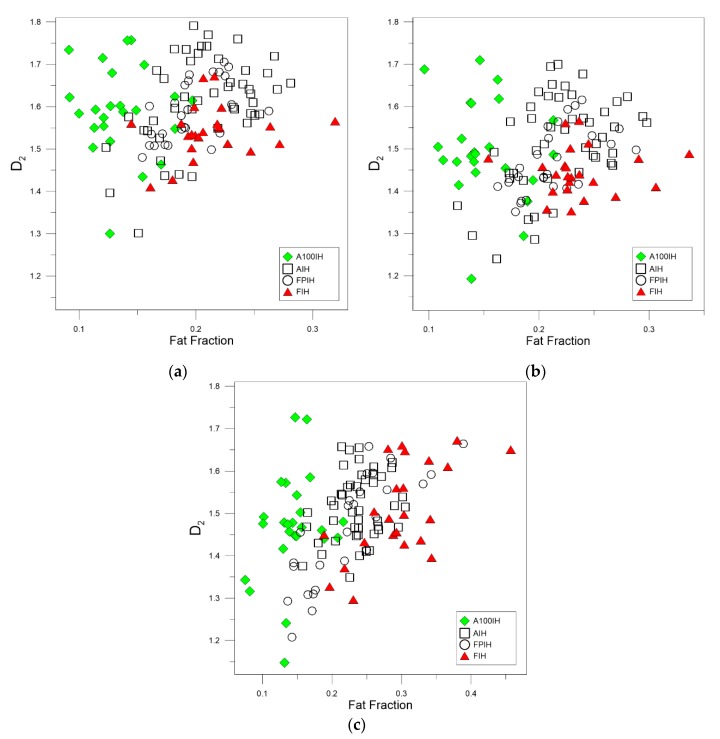
Scatter plots illustrating the relationships between the correlation dimension, *D*_2_, and the fat fraction obtained for each ham designation when (**a**) high-pass filter 25 pixels (**b**) high-pass filter 50 pixels (**c**) unfiltered images are used.

Figure 3, Figure 4 and Figure 5 show the relationship between the fractal dimensions and fat pixels in the image. For all these figures, plots (a) and (b) stand by high-pass filter 25 pixels and high-pass filter 50 pixels, respectively, while plots (c) correspond to unfiltered images. In the filtered methods analyzed, there is a clear relationship between the multifractal parameters and the fat fraction, as in the unfiltered method. As can be appreciated, the designations of A100IH and FIH are clearly separated in the three methods. These separations are not so evident in the denominations of AIH and FPIH.

The greatest variation observed between the high-pass filter methods and the unfiltered method is the dispersion of the points belonging to AIH in almost all the multifractal parameters.

**Figure 6:**
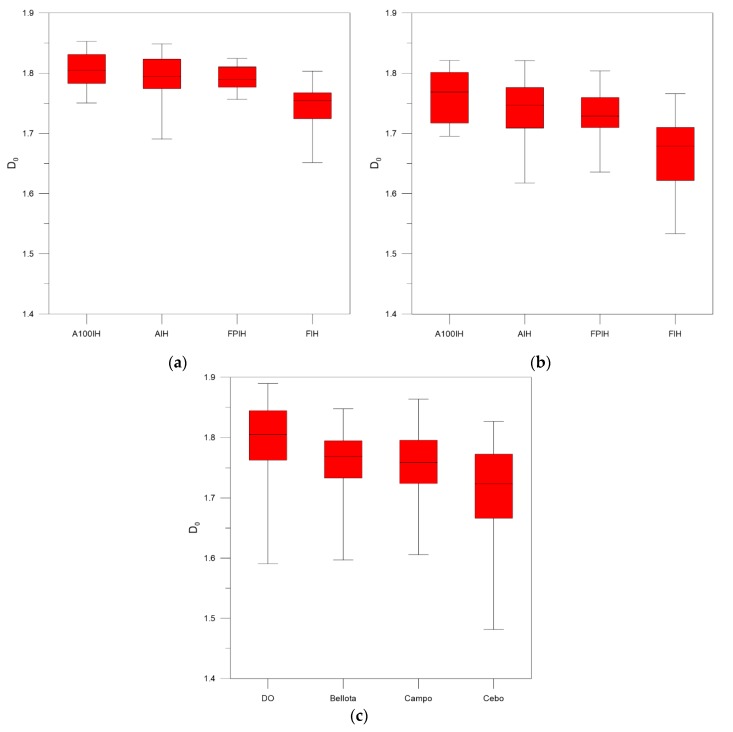
Box and whisker plots corresponding to the *D*_0_ fractal dimensions obtained for each ham designation from samples when (**a**) high-pass filter 25 pixels (**b**) high-pass filter 50 pixels (**c**) unfiltered images are used.

**Figure 7:**
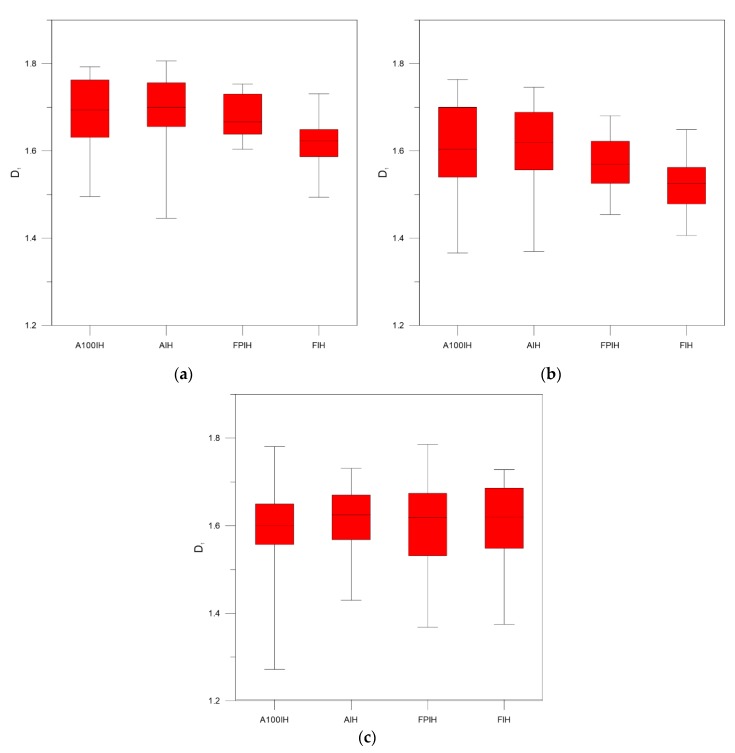
Box and whisker plots corresponding to the *D*_1_ fractal dimensions obtained for each ham designation from samples when (**a**) high-pass filter 25 pixels (**b**) high-pass filter 50 pixels (**c**) unfiltered images are used.

**Figure 8:**
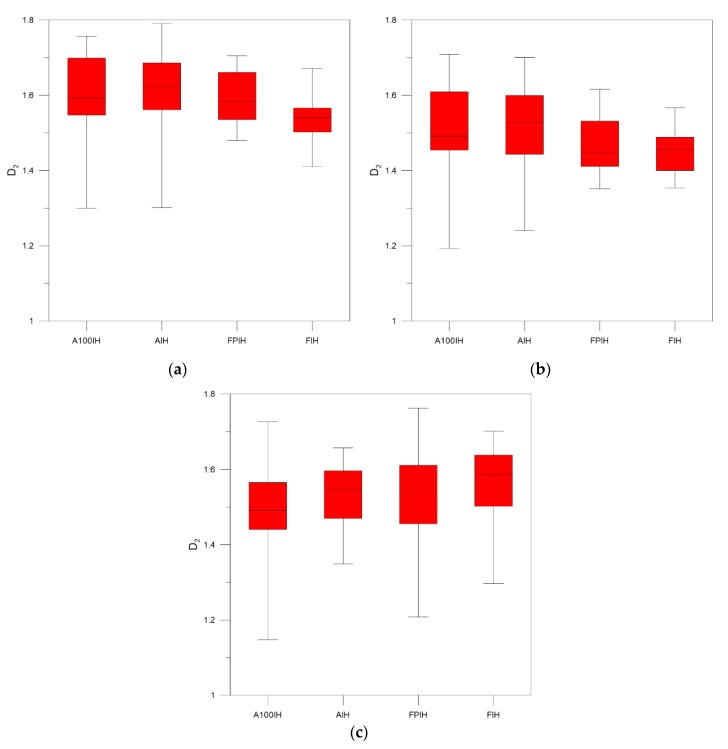
Box and whisker plots corresponding to the *D*_2_ fractal dimensions obtained for each ham designation from samples when (**a**) high-pass filter 25 pixels (**b**) high-pass filter 50 pixels (**c**) unfiltered images are used.

As can be seen in Figure 3, Figure 4 and Figure 5, when we compare the designations of A100IH and FIH separately, we can observe that there is a greater separation for parameters *D*_0_ and *D*_1_ and that the separation between them is greater for high-pass filters than for the unfiltered method. Figure 6, Figure 7 and Figure 8 represent the statistical distributions of the fractal dimensions (minimum, maximum, median, first quartile, and third quartile). As it happens to Figure 3, Figure 4 and Figure 5, plots (a), (b) and (c) in Figure 6, Figure 7 and Figure 8 show the results corresponding to high-pass filter 25 pixels, high-pass filter 50 pixels and unfiltered images, respectively. In general, the distributions are more grouped in filtering methods, which can establish a better discrimination of the results than the unfiltered method can.

In particular, there is a significant difference for A100IH and FIH on the *D*_0_, *D*_1_, and *D*_2_ parameters and, more specifically, for the 25 pixels radius method.

According to Figure 6, Figure 7 and Figure 8, the obtained results show the convenience of using high-pass filters because the error in the estimation of the multifractal parameters decreases compared to the unfiltered images. By means high-pass filters, the heterogeneity in the color of the muscle fibers is reduced, making the fractal dimensions distributions not as scattered as those obtained from the unfiltered images. A lower dispersion was found for the statistical distribution of the multifractal dimensions when high-pass filter 25 pixels was applied. This fact is especially evident for *D*_0_, and it is a consequence of dismissing the sensitivity of the multifractal analysis to signal noise by means of high-pass filters.

## 4. Conclusions

Quick methods to assess the quality of food and to detect food fraud offer numerous benefits for consumers and manufacturers. Nowadays, the determination of racial and feed designations of Iberian pigs requires expensive and slow methods that cannot be possibly applied online in slaughterhouses and cutting rooms.

The metrics derived from the multifractal-based image analysis of highly variable fat-connective tissue may be suitable for inclusion in the databases of predictive techniques of meat quality, such as data mining and machine learning. The multifractal approach extracts the information from the singularities detected for the image without applying any of the smoothing processes frequently used to transform discrete signals into continuous signals to compute their gradient. Therefore, no information is added or lost due to smoothing methods during the multifractal analysis. In addition, the independence of multifractal parameters over a range of scales and the fact that a specific data distribution is not required, as is the case when considering statistical methods, can be regarded as advantages of this approach. The digital food image analysis proposed in this work reduces the human bias, overcoming the sensitivity to noise shown by a multifractal analysis.

## Figures and Tables

**Table 1:** Number of samples and classification according to breed and nutrition.

Batch	A100IH	AIH	FPIH	FIH
1	12	26	12	11
2	13	21	14	12

A100IH: Acorn 100% Iberian Ham, AIH: Acorn Iberian Ham, FPIH: Feed/Pasture Iberian Ham, FIH: Feed Iberian Ham.

**Table 2:** The mean and standard error for the considered fractal dimensions in the four designations of Iberian ham for each method used.

Method	Ham Design	Fractal Dimension					
		*D*_0_		*D*_1_		*D*_2_	
		Mean	Standard Error	Mean	Standard Error	Mean	Standard Error
**Unfiltered Image**	A100IH	1.7991	0.0094	1.5967	0.0148	1.4885	0.0171
	AIH	1.7609	0.0051	1.6184	0.0067	1.5364	0.0078
	FPIH	1.7520	0.0075	1.6011	0.0131	1.5269	0.0165
	FIH	1.7101	0.0120	1.6055	0.0143	1.5589	0.0148
**High-Pass Filter 25 Pixels**	A100IH	1.8051	0.0048	1.6761	0.0146	1.5889	0.0209
	AIH	1.7939	0.0053	1.6906	0.0113	1.6140	0.0154
	FPIH	1.7914	0.0043	1.6760	0.0092	1.5875	0.0139
	FIH	1.7456	0.0079	1.6174	0.0113	1.5410	0.0128
**High-Pass Filter 50 Pixels**	A100IH	1.7650	0.0087	1.6021	0.0182	1.4978	0.0232
	AIH	1.7400	0.0071	1.6043	0.0135	1.5147	0.0163
	FPIH	1.7324	0.0071	1.5747	0.0119	1.4714	0.0151
	FIH	1.6666	0.0118	1.5193	0.0122	1.4456	0.0118
